# The quantitative trait linkage disequilibrium test: a more powerful alternative to the quantitative transmission disequilibrium test for use in the absence of population stratification

**DOI:** 10.1186/1471-2156-6-S1-S91

**Published:** 2005-12-30

**Authors:** Lorena M Havill, Thomas D Dyer, Dawn K Richardson, Michael C Mahaney, John Blangero

**Affiliations:** 1 Department of Genetics, Southwest Foundation for Biomedical Research San Antonio, TX, USA; 2 Department of Medicine, University of Texas Health Science Center San Antonio, TX, USA

## Abstract

Linkage analysis based on identity-by-descent allele-sharing can be used to identify a chromosomal region harboring a quantitative trait locus (QTL), but lacks the resolution required for gene identification. Consequently, linkage disequilibrium (association) analysis is often employed for fine-mapping. Variance-components based combined linkage and association analysis for quantitative traits in sib pairs, in which association is modeled as a mean effect and linkage is modeled in the covariance structure has been extended to general pedigrees (quantitative transmission disequilibrium test, QTDT). The QTDT approach accommodates data not only from parents and siblings, but also from all available relatives. QTDT is also robust to population stratification. However, when population stratification is absent, it is possible to utilize even more information, namely the additional information contained in the founder genotypes. In this paper, we introduce a simple modification of the allelic transmission scoring method used in the QTDT that results in a more powerful test of linkage disequilibrium, but is only applicable in the absence of population stratification. This test, the quantitative trait linkage disequilibrium (QTLD) test, has been incorporated into a new procedure in the statistical genetics computer package SOLAR. We apply this procedure in a linkage/association analysis of an electrophysiological measurement previously shown to be related to alcoholism. We also demonstrate by simulation the increase in power obtained with the QTLD test, relative to the QTDT, when a true association exists between a marker and a QTL.

## Background

Linkage analysis based on identity-by-descent (IBD) allele-sharing can be used to identify a chromosomal region harboring a quantitative trait locus (QTL), but lacks the resolution required for gene identification. Consequently, linkage disequilibrium (association) analysis is often employed for fine-mapping. Fulker et al. [1] proposed a variance-components based combined linkage and association analysis for quantitative traits in sib pairs, in which association is modeled as a mean effect and linkage is modeled in the covariance structure. To control for spurious associations due to population stratification and admixture, genotype scores are decomposed into between-pairs (**b**) and within-pairs (**w**) components. The resulting fixed effect model for the mean phenotype is

E [y] = μ + β_b_**b **+ β_w_**w**.

Because population structure will affect only the parameter β_b_, a robust test of association is obtained by comparing a model in which both β_b _and β_w _are freely estimated with a model in which β_w _is fixed at 0. Moreover, the presence of stratification can be inferred when the estimates of β_b _and β_w _differ significantly.

Abecasis et al. [2] extended this approach to general pedigrees. In their method, which is implemented in the program QTDT (quantitative transmission disequilibrium test) [3], genotypes are scored as the number of copies of the allele being tested minus 1. That is, an individual with no copies of the tested allele is assigned a -1, an individual with one copy of the allele receives a 0, and an individual with two copies is given a 1. Based on parental genotypes (or sibling genotypes, if parents are untyped), an expected genotype (**b**) is defined for each non-founder, and deviations (**w**) from this expectation are used to score allelic transmissions. For founders, **b **is set to the observed genotype and **w **is set to 0. As in the sib-pairs analysis described above, the model parameters associated with the between-family and within-family components of the genotype scores, β_b _and β_w_, are used to test for population stratification and linkage disequilibrium. Siegmund et al. [4] have proposed a similar method that incorporates an additional within pedigree/between family component for measuring population admixture. In contrast to QTDT, their method is based solely on the trait values for informative non-founders.

The QTDT approach accommodates data not only from parents and siblings, but also from all available relatives. This test is also robust to population stratification. However, when population stratification is absent, it is possible to utilize even more information, namely the additional information contained in the founder genotypes. In this paper, we introduce a simple modification of the allelic transmission scoring scheme of Abecasis et al. that results in a more powerful test of linkage disequilibrium, but is only applicable in the absence of population stratification. This test, the quantitative trait linkage disequilibrium (QTLD) test, has been incorporated into a new procedure in the statistical genetics computer package SOLAR [5]. Like the QTDT, the QTLD is a direct test of linkage disequilibrium whose type I error rate is not inflated in the presence of linkage. Thus, it can be used to partition the total evidence into independent linkage and linkage disequilibrium components. We apply this procedure in a linkage/association analysis of an electrophysiological measurement previously shown to be related to alcoholism. We also demonstrate by simulation the increase in power obtained with the QTLD test, relative to the QTDT, when a true association exists between a marker and a QTL.

## Methods

### The QTLD test

In the QTLD test, we model association as a fixed effect on the trait mean and we decompose the genotype scores into two components, **b' **and **w'**, where we have added primes to distinguish the QTLD components from those defined in the QTDT method. We modify the QTDT allelic transmission scoring in a simple way: founder genotypes are included in the within-family component rather than in the between-family component. That is, for founders, **w' **is set to the observed genotype and **b' **is set to 0. With this change in the scoring algorithm, our decomposition of the genotype scores no longer results in between- and within-family components, although we retain the notation for consistency.

The QTLD test procedure in SOLAR involves maximizing the likelihood of six genetic models:

Model 1: β_b _= 0, β_w _= 0 the association parameters are both fixed to 0

Model 2:  both parameters are estimated

Model 3:  the parameters are held equal

Model 4:  the within-family parameter is fixed to 0

Model 5:  both parameters are estimated

Model 6:  the regression parameter on **w' **is fixed to 0

In Models 1 through 4, the **b **and **w **components are defined according to the QTDT scheme, while in Models 5 and 6, the QTLD-defined components, **b' **and **w'**, are used. All models are derived from a previously maximized model which includes the trait of interest, any relevant covariates, and a linkage component based on IBD allele-sharing estimated from a microsatellite genome scan.

The following four tests are performed as part of the QTLD procedure:

1) Test for Stratification (Model 2 vs. Model 3) – The likelihood of a model in which the association parameters, β_b _and β_w_, are estimated is compared to the likelihood of a model in which they are constrained to be equal, as would be the case in the absence of population stratification.

2) Measured Genotype (Model 3 vs. Model 1) – This standard fixed effects regression [6] on the marker genotypes is used to test whether or not there is a significant difference between the genotypic means assuming additivity of allelic effects.

3) QTDT (Model 2 vs. Model 4) – This test is valid whether or not the stratification test indicates that population stratification is present.

4) QTLD (Model 6 vs. Model 5) – The QTLD test is only applicable in the *absence *of population stratification.

All test statistics are distributed as a χ^2 ^with one degree of freedom.

### Collaborative Study on the Genetics of Alcoholism (COGA) data

The data are from families participating in the Collaborative Study on the Genetics of Alcoholism (COGA) [7]. We restricted our analyses to non-Hispanic Whites to minimize population stratification. The dataset consists of 1,074 individuals in 119 pedigrees. All statistical genetic analyses were conducted using maximum likelihood-based variance decomposition approaches implemented in SOLAR.

We chose the TTTH1 electrophysiological endophenotype (electric potential FP1, far frontal left side channel) because the information supplied to Genetic Analysis Workshop participants regarding the COGA data indicated that microsatellite markers on chromosome 7 are linked to a QTL affecting normal variation in this phenotype. Linkage analyses were run at every 1-cM interval on chromosome 7 using multipoint estimates of IBD allele-sharing derived from the microsatellite marker data by the program LOKI [8,9]. Age at exam and maximum number of drinks consumed in a 24-hour period were included as covariates in the linkage analyses. COGA participants were not selected on the basis of the TTTH1 phenotype, hence no ascertainment correction was made. Subsequent to the linkage analysis, we used the single-nucleotide polymorphism (SNP) genotypes provided by Affymetrix and Illumina to conduct the QTLD test procedure in SOLAR, confining our analysis to that area of chromosome 7 showing evidence for linkage. For each SNP tested, we included a linkage component based on the short tandem repeat based multipoint IBDs for the integral centimorgan location nearest to the location of the SNP.

### Power to detect association

To demonstrate the increase in power that can be attained with the QTLD test, we conducted simulations to derive the expected χ^2 ^test statistic for each of the association tests (measured genotype, QTDT, and QTLD) given a true allelic association between a marker and a QTL. We used SOLAR to simulate a biallelic QTL responsible for up to 5% of the phenotypic variance for a quantitative trait having a total heritability of 30%. The family structure and pattern of missing data in the COGA dataset were assumed. We also simulated a fully informative marker completely linked to the QTL, which we used to calculate the IBD allele-sharing at the QTL location. Treating the QTL genotypes as if they had been observed for a marker in complete LD with the trait locus, we then ran the QTLD procedure, with a linkage component based on the IBDs included in the model, to obtain the χ^2 ^statistic for each of the tests. The test statistics were averaged over 100 replicates of the simulation for various values of the marker-specific heritability (i.e., the QTL effect size).

## Results and Discussion

### Linkage

**Figure 1:**
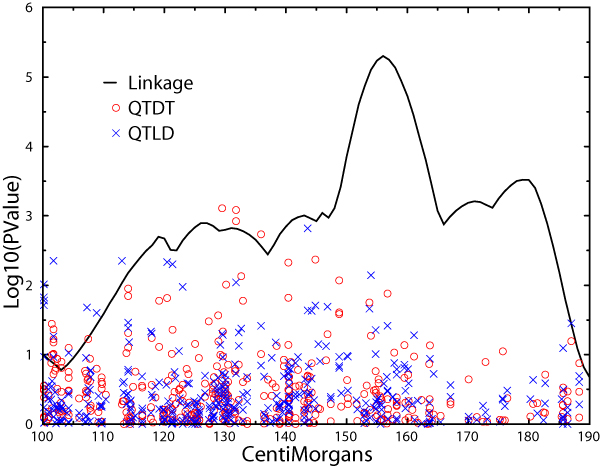
Results of linkage analysis based on microsatellite markers and of QTDT and QTLD linkage disequilibrium tests based on SNP genotypes.

The estimated heritability for TTTH1 was 0.31 ± 0.07 (*p *< 0.0001). The covariates age and max drinks accounted for approximately 15% of the phenotypic variance. A maximum LOD score of 4.24 was achieved at a point 156 cM from pter (see Figure 1). The region spanning 100 to 190 cM from pter exhibited evidence for linkage. A total of 395 SNPs (255 from Affymetrix and 140 from Illumina) fell into this region and were included in subsequent analyses.

### Stratification

Using a threshold of α = 0.05 in the stratification test described above, we found that 21 of the 395 SNPs showed evidence of population stratification. We had restricted our analysis to a single ethnic group in order to minimize the effects of population substructure, and this proportion (5.2%) of the total number of SNPs might have been expected to generate a significant test statistic purely by chance. Nevertheless, these SNPs were omitted from the QTLD portion of the test procedure.

### Association

Assuming 395 independent tests were performed, a 1 df χ^2 ^statistic of 14.69 (*p *= 0.000127) would be required for significant evidence of association. By this criterion, none of the tests, including the measured genotype test, showed significant evidence of association for any of the SNPs in the region of linkage. The Illumina SNP rs1896887, at location 129.5 cM, yielded the highest χ^2 ^value (11.296) for the QTDT, followed by the Affymetrix SNPs tsc0049494 (χ^2 ^= 11.179) and tsc0022400 (χ^2 ^= 10.507), both at a location 131.8 cM. The highest χ^2 ^value (10.0598) for the QTLD was observed for the Affymetrix SNP tsc0510163, at location 143.6 cM.

### Power

**Figure 2:**
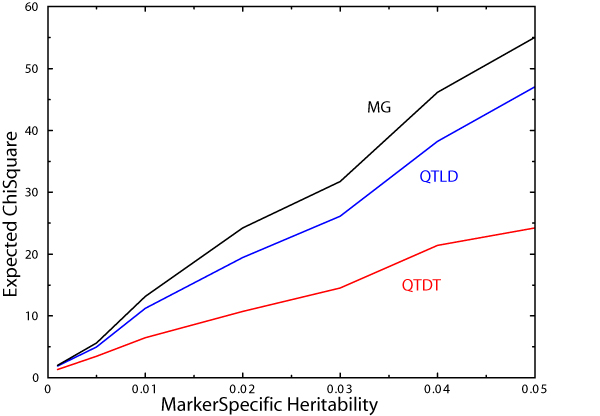
Expected χ^2 ^statistics for the measured genotype (MG), QTDT, and QTLD tests.

As shown in Figure 2, the QTLD test enjoys an advantage in power relative to the QTDT that increases as a function of the marker-specific heritability. Similarly, the classical measured genotype test uses even more of the relative association information and exhibits the most power. Although this would seem to suggest that the measured genotype would be the optimal test to use in the absence of population stratification it can be shown that for rare alleles, linkage alone can influence the measured genotype test (i.e., linkage can inflate the type I error rate for the test of linkage disequilibrium). In contrast, both the QTDT and the QTLD test are specific indicators of linkage disequilibrium between the marker and any causal variants. These results were obtained for a simulated minor allele frequency of 0.25. Simulations with an allele frequency of 0.1 yielded similar results (not shown), indicating that the difference in power is not primarily a function of the marker allele frequencies. Note that the marker-specific heritability is proportional to the QTL effect size, where the constant of proportionality is equal to the square of the correlation (i.e., LD) between the marker and the trait locus; therefore, the results obtained for the complete LD case should be directly relevant to the case of incomplete LD.

## Conclusion

We have introduced the QTLD test, a novel approach for detecting association due to linkage disequilibrium in the absence of population stratification. We have confirmed in our simulations that the QTLD test provides a significant increase, relative to the QTDT, in power to detect an allelic association. Investigators can now use SOLAR to conduct a combined linkage and association analysis, using pedigrees of arbitrary size and complexity, that includes a test for population stratification along with several tests of association: measured genotype, QTDT, and, where appropriate, the QTLD test. Our application of this procedure to the COGA data for TTTH1 identified several SNPs under the chromosome 7 linkage peak which exhibit suggestive levels of association, although none were statistically significant after correction for multiple testing.

## Abbreviations

COGA: Collaborative Study on the Genetics of Alcoholism

IBD: Identity by descent

QTL: Quantitative trait locus

QTDT: Quantitative transmission disequilibrium test

QTLD: Quantitative trait linkage disequilibrium

SNP: Single-nucleotide polymorphism

## Authors' contributions

JB, LMH, and TDD developed the QTLD methodology. LMH, with the assistance of DKR and TDD, performed the analysis of the COGA data and drafted the manuscript. TDD conducted the power simulations. MCM provided a critical review of the methodology. All authors have read and approved this manuscript.
